# An Individualized Low-Intensity Walking Clinic Leads to Improvement in Frailty Characteristics in Older Veterans

**DOI:** 10.14283/jfa.2019.16

**Published:** 2019-05-30

**Authors:** Sara E. Espinoza, B. Orsak, C.-P. Wang, D. Maccarthy, D. Kellogg, B. Powers, A. Conde, M. Moris, P.R. Padala, K.P. Padala

**Affiliations:** 1 Department of Medicine, Division of Geriatrics, Gerontology & Palliative Medicine, University of Texas Health Science Center at San Antonio, San Antonio, USA; 2 Sam and Ann Barshop Institute for Longevity and Aging Studies, University of Texas Health Science Center at San Antonio, San Antonio, USA; 3 Geriatric Research, Education & Clinical Center (GRECC), South Texas Veterans Health Care System, San Antonio, USA; 4 Department of Epidemiology and Biostatistics, University of Texas Health Science Center at San Antonio, San Antonio, USA; 5 GRECC, Central Arkansas Veterans Healthcare System, Little Rock, USA; 6 Department of Psychiatry, University of Arkansas for Medical Sciences, Little Rock, USA; 7 Department of Geriatrics, University of Arkansas for Medical Sciences, Little Rock, USA; 8 Mail Code 7875, 7703 Floyd Curl Drive, 78223, San Antonio, TX, USA

**Keywords:** Frailty, physical activity, gait speed

## Abstract

**Background:**

Sedentary lifestyle leads to worse health outcomes with aging, including frailty. Older adults can benefit from regular physical activity, but exercise promotion in the clinical setting is challenging.

**Objectives:**

The objective of this clinical demonstration project was to implement a Geriatric Walking Clinic for older adults and determine whether this clinical program can lead to improvements in characteristics of frailty.

**Design:**

This was a clinical demonstration project/quality improvement project.

**Setting:**

Outpatient geriatrics clinic at the South Texas Veterans Health Care System (STVHCS).

**Participants:**

Older Veterans, aged ≥60 years.

**Intervention:**

A 6-week structured walking program, delivered by a registered nurse and geriatrician. Patients received a pedometer and a comprehensive safety evaluation at an initial face-to-face visit. They were subsequently followed with weekly phone calls and participated in a final face-to-face follow-up visit at 6 weeks.

**Measurements:**

Grip strength (handheld dynamometer), gait speed (10-ft walk), Timed Up and Go (TUG), and body mass index (BMI) were assessed at baseline and follow-up. Frailty status for gait speed was assessed using Fried criteria.

**Results:**

One hundred eighty five patients completed the program (mean age: 68.4 ±7 years, 88% male). Improvements from baseline to follow-up were observed in average steps/day, gait speed, TUG, and BMI. Improvement in gait speed (1.13 ±0.20 vs. 1.24 ± 0.23 meter/second, p<0.0001) resulted in reduced odds of meeting frailty criteria for slow gait at follow-up compared to the baseline examination (odds ratio = 0.31, 95% confidence interval: 0.13–0.72, p = 0.01).

**Conclusions:**

Our findings demonstrate that a short duration, low-intensity walking intervention improves gait speed and TUG. This new clinical model may be useful for the promotion of physical activity, and for the prevention or amelioration of frailty characteristics in older adults.

## Introduction

Over the past two decades, the geriatrics community has recognized frailty as a geriatric syndrome that dramatically affects physical function, quality of life, and leads to increased health care costs. The estimated cost of frailty in the U.S. was over $18 billion in 2000 (1). Several studies have shown that frailty leads to falls, hospitalization, disability, and an increased risk of nursing home placement (2, 3, 4). Older adults are prone to adopt a sedentary lifestyle with increasing age (5), which can contribute to poor health and disability (6). Low physical activity is one of the key frailty characteristics in the Fried frailty model; others include unintentional weight loss, slow gait, muscle weakness, and self-reported exhaustion (2, 7). Given the enormous costs associated with frailty, both personal and economic, a clinical intervention that prevents or delays frailty that is easy to deliver in a clinical setting would have a major impact on our society.

Exercise is known to have major health benefits for older adults, including improvement in frailty-related measures, such as gait speed, and improvement in the ability to perform activities of daily living (8, 9). Even the most frail older adults can have significant improvements in strength and physical function with exercise (10). While the benefits of exercise are known, few clinical trials have focused on the Veteran population, and few studies have examined the feasibility of implementing a physical activity intervention in a clinical setting. We do know, however, that engagement and regular contact with clinical staff and physicians along with an exercise prescription can have a greater impact on future exercise success (11, 12).

To address this and to promote an active lifestyle in older Veterans at South Texas Veterans Health Care System (STVHCS), we developed an outpatient intervention to promote physical activity in older Veterans. This intervention was modeled after the Little Rock Geriatric Walking Clinic (GWC) while maintaining a specific focus of the local site which was to improve components of frailty (13). The Little Rock GWC is a patient-centric program that implements a comprehensive approach to engage older Veterans in a long-term program of regular physical activity primarily in the form of walking. This clinic uses proven strategies, such as motivational counseling, follow-up phone calls from a nurse, and self-monitoring using pedometers. Here we report short-term results of this program from the 6-week follow-up.

## Methods

### Patient Population

The Geriatric Walking Clinic is a 6-week walking program designed to encourage increased physical activity in older adults through regular walking. Patients from the outpatient services at STVHCS were eligible for participation if they were 60 years or older, willing to walk for exercise, and willing to accept weekly phone calls. Patients were recruited via: 1) flyers/brochures placed in patient waiting areas within the main hospital (Audie L. Murphy Memorial VA Hospital) and Community-based Outpatient Clinics (CBOCs) within STVHCS; 2) community events and health fairs for Veterans within STVHCS; 3) an electronic kiosk placed in the Geriatric Evaluation and Management (GEM) Clinic waiting room with electronic flyers; and 4) referrals from primary care providers (PCPs) through a consult within the electronic medical record system (CPRS), which was available to all PCPs at STVHCS. This project was approved as non-research/quality improvement by the Institutional Review Board at the University of Texas Health Science Center at San Antonio, which serves the STVHCS, and consent was not required.

### Patient Screening

Patients were evaluated for safety for walking exercise based on the National Institute of Aging's (NIA) Exercise Assessment and Screening for You (EASY) criteria (14) and additional medical contraindications. The NIA EASY is a six-item screening tool that helps in selecting older adults for safe participation in an exercise program. The tool includes questions regarding chest pain and tightness during physical activity, dizziness, high blood pressure, pain, stiffness and swelling of joints, falls or feeling unsteady while walking, or any other reason the patient would be concerned about starting a physical activity program. Specific medical contraindications were unstable angina, severe left main coronary artery disease, end stage congestive heart failure (ejection fraction <30%), severe aortic valvular disease, uncontrolled cardiac arrhythmia, uncontrolled hypertension (systolic >180 mmHg or diastolic >100 mmHg), large abdominal aortic aneurysm, severe shortness of breath, cognitive impairment that interferes with compliance, stroke within the prior six months, uncontrolled diabetes mellitus (Hemoglobin A1c [HbA1c] >10%), and pain limiting walking. All subjects were seen and evaluated by a geriatrician who reviewed their medical history and performed an evaluation to determine appropriateness for participation in the program.

### Clinical Intervention

The clinic involved an initial baseline face-to-face visit, weekly telephone follow-up calls during the period of the intervention, and a follow-up 6-week face-to-face visit. At the baseline clinic visit, patients met individually with a registered nurse (RN) (who was also a certified diabetes educator) and a geriatrician to screen for eligibility and safety for regular walking, perform baseline assessments (see Table 1), support the patient in self-setting individualized goals for the program, issue a pedometer, and train the patient on the use of the pedometer. Patients also received individualized counseling on healthy food intake along with educational materials about benefits of walking at the baseline visit.

**Table 1:** Baseline and follow-up assessments in the Geriatrics Walking Clinic

Assessment	Baseline	Follow-up
Blood pressure, height, weight	X	X
Cognitive assessment using either CLOX or Mini Cog^a^	X	
Unintentional weight loss, physical activity, fatigue^b^	X	
Gait Speed, grip strength, timed up and go test	X	X


a. To exclude patients with significant cognitive impairment from participating in the clinic; b. To establish the diagnosis of frailty at baseline

During the first week of the program, patients were instructed to perform usual activities and not to change their routine, in order to establish a baseline average step count per day. Weekly telephone calls were made by the RN to inquire about daily step counts; troubleshoot barriers to walking; offer suggestions, encouragement, and counseling; and help the patient establish a goal for an increase in daily step counts (targeting a 5–10% increase each week).

At approximately 6 weeks, depending on patient availability, patients were seen for a face-to-face visit for review of progress and re-assessment of physical measurements. In order to facilitate continued lifestyle change and walking for exercise, patients were encouraged and provided education on Veterans Administration (VA) and community resources available for continued exercise. At the follow-up visit, patients were encouraged to continue to walk and monitor using their pedometers, received an additional phone call one month later, and received a certificate of completion.

### Assessments

Several assessments were measured at either the baseline, follow-up, or both, as shown in Table 1. At baseline, components of frailty measured included unintentional weight loss, usual levels of physical activity (15), self-reported exhaustion, Timed Get Up and Go (TUG), gait speed, and grip strength. The Fried frailty phenotype criteria (3) were used to determine whether patients met standardized criteria for the physical characteristics of frailty, including weakness (grip strength) and slowness (10-foot walk). The cut-points used were standardized in the San Antonio Longitudinal Study of Aging, which is ethnically similar to our clinic population (7) and are provided in Appendix Table 1. The TUG and gait speed measured lower extremity strength at baseline and follow-up (16). Follow-up measurements of exhaustion and physical activity were not conducted. Therefore, change in meeting frailty classification could not be assessed for exhaustion or physical activity. Although weight loss with the intervention was assessed, unintentional weight loss per se would not be expected to change within the time frame of the intervention. Therefore, formal assessment of change in frailty criteria from baseline to follow-up was conducted for gait speed and grip strength only. A brief evaluation of cognitive function was assessed at the baseline clinic visit using either the CLOX (17) or the Mini Cog (18) assessments to exclude participants with severe cognitive impairment that may hinder their participation.

### Statistical Analysis

Summary statistics were used to characterize the patient population. Change in steps taken per day, gait speed, TUG, BMI, and grip strength from baseline to follow-up at 6 weeks was examined using paired t-tests. To account for correlation of repeated measures, the generalized estimating equation (GEE) technique assuming a binomial distribution with the logit link was used to determine the changes in frailty classification (not frail vs. frail) in slowness and weakness based on walking speed and grip strength from baseline to the end of the program. Both the unadjusted GEE analysis (or asymptotic unconditional McNemar test)[19] and the adjusted results are reported. The covariates included in the adjusted model are age, education, diabetes, and baseline physical activity.

## Results

Patient characteristics are shown in Table 2. Mean age (±SD) was 68.4 ±6.8 years, 88% were male, and the majority were Hispanic (42.7%). The majority of the patients were obese (61.8%) and had diabetes (55.7%). Follow-up information is provided only for those who completed the follow-up assessments (N = 157); 28 individuals examined at baseline did not complete the follow-up exam (15%). There were no major side effects or injuries that occurred as a result of the intervention. As shown in Table 3, from baseline to follow-up we observed a significant increase in the number of steps taken per day and significant improvements in gait speed (improved by 0.096 ±0.017 meter/second), TUG (improved by 0.8 second), and BMI (improved by 0.3 kg/m2). The average number of steps per day after the intervention increased by 1,426 (p <0.001) steps in the unadjusted analysis, and by 1,523 steps (p <0.001) in the adjusted analysis. No significant change was observed in grip strength from baseline to follow-up. Thirteen patients met the frailty criterion of low gait speed at baseline. Nearly 85% (N=11) of them improved their gait speed sufficiently so as to not meet the frailty criteria at the 6-week follow-up visit. Improvement in gait speed (1.13 ±0.20 vs. 1.24 ±0.23 meters/second, p <0.0001) resulted in reduced number of patients meeting criteria for frailty at the follow-up (10.3% met criteria at baseline vs. 3.5% met criteria following the intervention). This resulted in reduced odds of meeting frailty criteria for slow gait at follow-up compared to the baseline examination with an unadjusted odds ratio (OR) of 0.31, 95% confidence interval (CI): 0.13–0.72, p =0.0062. After adjustment for covariates, the adjusted OR was 0.26, 95% CI: 0.10–0.69, p =0.0063. This suggests a 74% reduced odds of meeting frailty criteria for gait speed. There was limited, non-significant improvement in grip strength at the follow-up compared to the baseline (31.7 ±9.8 kg vs. 31.2 ±9.9 kg, p =0.1765). This resulted in 37.2% meeting frailty criteria for grip strength post-intervention compared to 38.5% meeting frailty criteria at baseline. The odds of meeting frailty criteria for grip strength at follow-up compared to the baseline examination was not significant in either the unadjusted model (OR = 0.83, p =0.147) or the adjusted model (OR = 0.81, p =0.149).

**Table 2:** Patient characteristics (N = 185)

	Mean (SD) or n (%)
Age, years (range: 52–89)	68.4 (6.8)
Male sex	163 (88.0)
Ethnic group: Hispanic	79 (42.7)
Non-Hispanic White	75 (40.5)
African American	27 (14.6)
Education, years, (range: 5–20)	13.8 (2.5)
Body mass index (BMI), kg/m2, (range: 19.9 – 52.9 kg/m^2^)	32.2 (5.6)
Body mass index category	
Normal (BMI 18.5 – 24.9 kg/m2)	11 (5.9)
Overweight (BMI 25 – 29.9 kg/m2)	60 (32.3)
Obese (BMI ≥ 30 kg/m2)	115 (61.8)
Tobacco use, current	12 (6.5)
Hypertension	150 (81.1)
Diabetes	103 (55.7)
Coronary artery disease	40 (21.6)
Stroke	6 (3.2)
Congestive heart failure	11 (5.9)
Fallen in the last year	64 (34.6)
Physical activity, kcal/week, (range: 0–16124.2)	4605.0 (3317.5)
Timed up and go, seconds, (range: 6.7–19.5)	10.5 (2.4)
Walking speed (10 foot walk), meters/second, (range: 0.65–1.69)	1.13 (0.20)
Grip strength, kg, (range: 9.4–59.3)	31.6 (9.6)

**Table 3:** Change in characteristics from baseline to follow-up at 6 weeks (N =157)

	Baseline Mean (SD)	Follow-upMean (SD)	ChangeMean (SD)	P-value
Steps per day, number	4302 (2716)	5728 (3336)	1426 (2687)	<0.0001
Body mass index, kg/m^2^	32.1 (5.2)	31.8 (5.2)	−0.3 (1.1)	0.0011
10-foot walk, meters/second	1.13 (0.20)	1.24 (0.23)	0.096	<0.0001
Timed Up and Go, seconds	10.3 (2.3)	9.5 (2.0)	−0.8 (1.2)	<0.0001
Grip strength, kg	31.2 (9.9)	31.7 (9.8)	0.5 (4.2)	0.1765

## Discussion

Our results demonstrate the feasibility of a low-impact activity promotion clinic. We also demonstrate that a clinic that promotes walking as physical activity in older adults leads to improvements in gait speed, TUG, and BMI even at a short follow-up of six weeks. The improvement in gait was associated with reduction in the proportion of individuals who met gait speed criteria for frailty from baseline to follow-up.

The improvement in gait speed is also clinically significant, and it is expected that these changes will translate, on the long term, into clinically relevant improved outcomes (20). Prior studies have demonstrated the potential usefulness and effectiveness of exercise for the promotion of healthy aging, prevention of frailty (21, 22, 23, 24, 9, 25), and for amelioration of frailty in those who were already frail (8, 26). However, the results of the prior studies were mixed which may be partially related to differing follow-up periods as well as methods used for assessing frailty across studies. The Lifestyle Interventions and Independence for Elders (LIFE) study examined the effectiveness of a physical activity intervention in sedentary older adults who were at increased risk for future disability. Initial exploratory findings suggested that the intervention, which included aerobic, strength, flexibility, and balance training, led to improvement in frailty (measured by Fried criteria) (3) over one year follow-up, primarily due to an increase in physical activity in the intervention group (21). Later analysis from this study demonstrated that there was no difference in frailty (measured using an abbreviated frailty criteria) (27) from baseline to follow-up (22). In spite of these conflicting results, the general consensus remains that physical activity is the main intervention for frailty (9).

Our study has some weaknesses, including the fairly small number of patients evaluated, a short follow-up period, and that this was not a randomized controlled trial. We also emphasize that even though individuals improved in frailty on one of the frailty characteristics (walking speed), this may not necessarily reflect a global change in frailty classification (i.e., non-frail, pre-frail, or frail). Further, because the patients referred to the Geriatric Walking Clinic were either self-referred or were referred by their treating PCP, we can only observe the effect of this clinical intervention in motivated individuals, many of them who also have support from their doctor. Further, individuals with medical conditions that would limit safety with exercise were excluded. Therefore, this sample includes relatively healthy older adults. Here, we present the results of a quality improvement program; therefore, a strength of our evaluation is that we have demonstrated the effect of promoting walking for exercise in a “real world” clinical setting. Robust representation of minority participants is a significant strength of our program. Similarly, high rates of diabetes could be seen as a strength as patients with diabetes have higher sedentary behavior.

Prior work has demonstrated that exercise is beneficial for older adults. A meta-analysis which conducted a combined analysis of 1,068 participants in eight clinical trials demonstrated that exercise led to improvement in gait speed, balance, and disability in activities of daily living (8). Current exercise recommendations for older adults include 150 minutes of moderate-intensity aerobic activity (such as brisk walking) each week and muscle strengthening exercises on two or more days per week (28). Practically speaking, however, the majority of older adults are highly sedentary, and these recommendations may be an ambitious goal and difficult to achieve in the clinical setting with other competing needs. In fact, among adults aged 65 to 74 years, only 34% of men and 17% of women expend more than 2,000 kcal per week in exercise (29). Physical inactivity and health consequences cost over 11% of US healthcare expenditures, about $117 billion in 2014 alone (30). Further, although the exercise recommendations above are a reasonable starting point, several exercise programs with varied approaches, frequency and duration have led to improvements in muscle strength, gait speed, balance, and falls reduction in older adults (31). Therefore, the optimal exercise program for older adults with varying needs is not always clear and likely depends upon a patient's comorbid medical conditions as well as desired results. Current evidence does support that exercise which is delivered in a prescribed manner concludes in improved results (11, 12). Our program adds to the existing literature by showing the feasibility and outcomes of a low impact walking prescription wherein the goals were negotiated with the patient, and ongoing motivational support was provided with weekly phone calls. Improvements in gait speed may have a lasting benefit on individual patients' longevity (32). Best et al., in a recent paper, showed that early change in walking speed was predictive of improvement in chronic physical activity among a group of over 2800 community dwelling older adults, further emphasizing the lasting health effects of the increased gait speed found in this study (33).

In summary, our Geriatric Walking Clinic encourages walking for exercise in a “start low and go slow” manner (a familiar tenet in the practice of geriatric medicine) with frequent monitoring and encouragement. Our findings demonstrate that a low-intensity walking intervention leads to improvements in gait speed, one of the frailty criteria for slowness even at a short follow-up. This new clinical model may be useful for the promotion of physical activity and for the prevention or amelioration of frailty in older adults.

*Funding:* Funded by VA T21 Non-Institutional Long Term Care Initiative grant “Activity Promotion Consultation Clinic to Engage High-risk Older Veterans in Regular Walking” (VISN 17-G598-4), VA ORH grant “Geriatrics Walking Clinic: Rural Expansion” (VA ORH S2-P00738), and the San Antonio Older Americans Independence Center (NIA/NIH P30 AG044271).

*Conflict of Interest:* There are no conflicts of interest

*Ethical standard:* This article details results from a clinical demonstration project and does not contain any study involving human subjects.

## Electronic Supplementary Material

Appendix Table 1. Standardization Subgroups and Frailty Cutoffs for Walking Speed and Grip Strength
