# Supplementary material for: An Individualized Low-Intensity Walking Clinic Leads to Improvement in Frailty Characteristics in Older Veterans
Source: J Frailty Aging. 2019 May 30;8(4):205–9. doi: 10.14283/jfa.2019.16 (PMC12275737; doi:10.14283/jfa.2019.16)
Supplement: Supplementary file 1 — Appendix Table 1. Standardization Subgroups and Frailty Cutoffs for Walking Speed and Grip Strength [file mmc1.pdf]

**Appendix Table 1. Standardization Subgroups and Frailty Cutoffs for Walking Speed and Grip Strength**		
**Walking speed**	**Median height (inches) subgroups**	**Frailty cutoff (seconds)**
Men	< 67.25	≥ 4.19
	≥ 67.25	≥ 3.65
Women	< 61.88	≥ 5.31
	≥ 61.88	≥ 4.25
**Grip strength**	**BMI quartile (kg/m^2) subgroups**	**Frailty cutoff (kg)**
Male	≤ 25.1	< 29.4
	25.2 – 27.3	< 30.5
	27.4 – 29.8	< 32.3
	> 29.8	< 29.1
Female	≤ 25.0	< 19.3
	25.1 – 28.1	< 18.5
	28.2 – 31.7	< 17.6
	> 31.7	< 17.7
